# Gut Dysbiosis in Patients with Anorexia Nervosa

**DOI:** 10.1371/journal.pone.0145274

**Published:** 2015-12-18

**Authors:** Chihiro Morita, Hirokazu Tsuji, Tomokazu Hata, Motoharu Gondo, Shu Takakura, Keisuke Kawai, Kazufumi Yoshihara, Kiyohito Ogata, Koji Nomoto, Kouji Miyazaki, Nobuyuki Sudo

**Affiliations:** 1 Department of Psychosomatic Medicine, Graduate School of Medical Sciences, Kyushu University, Fukuoka, Japan; 2 Yakult Central Institute, Tokyo, Japan; GI Lab, UNITED STATES

## Abstract

Anorexia nervosa (AN) is a psychological illness with devastating physical consequences; however, its pathophysiological mechanism remains unclear. Because numerous reports have indicated the importance of gut microbiota in the regulation of weight gain, it is reasonable to speculate that AN patients might have a microbial imbalance, i.e. dysbiosis, in their gut. In this study, we compared the fecal microbiota of female patients with AN (n = 25), including restrictive (ANR, n = 14) and binge-eating (ANBP, n = 11) subtypes, with those of age-matched healthy female controls (n = 21) using the Yakult Intestinal Flora-SCAN based on 16S or 23S rRNA–targeted RT–quantitative PCR technology. AN patients had significantly lower amounts of total bacteria and obligate anaerobes including those from the *Clostridium coccoides* group, *Clostridium leptum* subgroup, and *Bacteroides fragilis* group than the age-matched healthy women. Lower numbers of *Streptococcus* were also found in the AN group than in the control group. In the analysis based on AN subtypes, the counts of the *Bacteroides fragilis* group in the ANR and ANBP groups and the counts of the *Clostridium coccoides* group in the ANR group were significantly lower than those in the control group. The detection rate of the *Lactobacillus plantarum* subgroup was significantly lower in the AN group than in the control group. The AN group had significantly lower acetic and propionic acid concentrations in the feces than the control group. Moreover, the subtype analysis showed that the fecal concentrations of acetic acid were lower in the ANR group than in the control group. Principal component analysis confirmed a clear difference in the bacterial components between the AN patients and healthy women. Collectively, these results clearly indicate the existence of dysbiosis in the gut of AN patients.

## Introduction

Eating disorders are an important cause of physical and psychosocial morbidity in adolescent girls and adult women [1]. Anorexia nervosa (AN), a type of eating disorder, is classified into two main sub-types. Restricting type (ANR) is the most common type of AN in which a patient severely restricts his/her food intake. Patients with binge-eating or purging type AN (ANBP) restrict their intake and also have abnormal eating or purging behaviors, such as self-induced vomiting.

In AN patients, the energy requirement for body-weight gain is higher than would be expected because of the cost of energy storage [2–4]. Factors such as increased physical activity [5] or diet-induced thermogenesis [6] are potentially involved in the poor weight gain response; however, the precise mechanism explaining this discrepancy remains to be clarified.

Gut microbiota not only play a critical role in the development of the gut mucosal immunity [7, 8], but also affect the regulation of the hypothalamic-pituitary-adrenal (HPA) axis [9] and additional behaviors [10–14]. With the recent number of reports describing the importance of gut microbiota in the regulation of weight gain and host adiposity [15–17], it is reasonable to speculate that AN patients might have a microbial imbalance in their gut, i.e. gut dysbiosis.

In the current study, we compared the fecal microbiota of female AN patients with those of age-matched healthy female controls, using the Yakult Intestinal Flora-SCAN (YIF-SCAN^®^) based on 16S or 23S rRNA–targeted RT–quantitative PCR (RT-qPCR) technology.

## Materials and Methods

### Subjects

We enrolled Japanese AN patients who were admitted to our department or visited our outpatient section at Kyushu University Hospital between 2010 and 2013, and 25 female patients (14 ANR and 11 ANBP) agreed to participate in this study. We also enrolled 21 age-matched, healthy female volunteers. Volunteers with a history of digestive disease such as inflammatory bowel disease and irritable bowel syndrome were excluded. We also excluded participants with the following conditions from the study: severe physical diseases, such as renal failure and infectious diseases and a history of antibiotics use or a regular intake of yoghurt or probiotics within 3 months of study participation.

The AN patients underwent a structured interview, and their current AN phenotypes were diagnosed according to the Diagnostic and Statistical Manual of Mental Disorders-IV-TR criteria. The study protocol was approved by the Institutional Review Boards of Kyushu University Hospital, and written informed consent was obtained from all of the participants before enrollment in the study.

### Biochemical Analysis of Blood Samples

Blood samples were collected in the morning for determination of serum levels of albumin, blood urea nitrogen, creatinine, electrolytes, aspartate aminotransferase, alanine aminotransferase, total cholesterol, triglyceride, and glucose. High sensitivity C-reactive protein levels were determined by latex nephelometry.

### Bacterial Count by Yakult Intestinal Flora-SCAN (YIF-SCAN^®^)

Fecal samples were collected from the participants, according to a method described previously [18]. Then, we extracted total RNA fractions from feces using a method that has also been described previously and examined the composition of major gut bacterial groups using the YIF-SCAN^®^ based on 16S or 23S rRNA–targeted RT–qPCR technology [18–23]. The specificity of the RT-qPCR assay, the sequences of the primers, and the lower detection limit for each bacterium were thoroughly checked and determined (S1 Table), as previously described [18–23]. The detection rate of each bacterium was evaluated in individual groups by calculating the ratio of the number of individuals who had the bacterium to the total number of individuals in the group.

### Organic Acids and pH Levels in Feces

We measured fecal organic acids according to methods described previously [22]. In brief, the fecal sample was homogenized in perchloric acid (0.15 mol/L), and the resulting suspension was collected by centrifugation at 20,400× *g* at 4°C for 10 min. The concentrations of organic acids in the sample were measured using a high-performance liquid chromatography system (432 Conductivity Detector; Waters Co., Milford, MA). We also checked fecal pH using the IQ 150 pH/Thermometer (IQScientific Instruments, Inc., Carlsbad, CA).

### Statistical Analysis

All data are expressed as the mean ± SD. All analyses were performed using the JMP statistical software package for Windows (version 11.0.0, SAS Institute Japan). To evaluate differences between the control subjects and AN patients, we used the Wilcoxon-Mann-Whitney test followed by the Bonferroni correction based on the number of tests. To examine differences in serum chemical parameters and bacterial counts between the control, ANR, and ANBP groups, the nonparametric Kruskal-Wallis test was used. Comparisons between two groups (control vs. ANR, control vs. ANBP, or ANR vs. ANBP) were performed using the Wilcoxon-Mann-Whitney test followed by the Bonferroni correction based on the number of tests.

The distribution of the standardized *U* value is close to the normal distribution when sample sizes are not too small [24]. In that case, the standardized value (Z-score) is given by the following equation:

$$\text{Z}=\frac{U-m_{U}}{\sigma_{U}}$$

The *m*_*U*_ and *σ*_*U*_ are the mean and standard deviation of *U*, respectively:

$$m_{U}=\frac{n_{1}n_{2}}{2}$$

$$\sigma_{U}=\sqrt{\frac{n_{1}n_{2}(n_{1}+n_{2}+1)}{12}}$$

The effect size (*r*) was calculated using absolute value of the Z-score and N (total number of paired participants in the groups), according to the following equation:

$$r=\frac{z}{\sqrt{N}}$$

According to previous literature for *r*, a value of 0.5 represents a large effect; 0.3, a medium effect; and 0.1, a small effect [24, 25].

Regarding the detection rate of each bacterium, comparisons between two groups were analyzed using the Fisher’s exact test and subjected to the Bonferroni correction based on the number of tests.

For principal component analysis (PCA), the log-transformed bacterial count was used. For samples in which bacteria were not detected (ND), the bacterial counts were regarded to be half the detection limits of the corresponding primer sets (S1 Table). PCA was applied to determine the data sets by using the statistical programming language R 3.1.1 [23]. The following data were included in the analysis: total bacteria, *Clostridium coccoides* group, *Clostridium leptum* subgroup, *Bacteroides fragilis* group, *Bifidobacterium*, *Atopobium* cluster, *Prevotella*, *Enterobacteriaceae*, *Enterococcus*, *Staphylococcus*, *Streptococcus*, *Clostridium difficile*, *Clostridium perfringens*, and total *Lactobacillus*. The result of the PCA was visualized using the ade4 package provided in the program R 3.1.1.

## Results

As shown in Table 1, weight, body mass index, and serum C-reactive protein levels were significantly lower in the AN group than in the control group. In contrast, the AN group had significantly higher serum aspartate aminotransferase and alanine aminotransferase levels than the control group. In the analysis based on AN subtypes (Table 2), weight and body mass index in both the ANR and ANBP groups were significantly lower than those in the control group. In contrast, serum aspartate aminotransferase levels in both AN subtype groups were significantly higher than those in the control group. The ANBP group had significantly lower serum levels of potassium than the control and ANR groups. The ANR group exhibited significantly lower serum levels of C-reactive protein than the control group. In contrast, serum alanine aminotransferase levels were significantly higher in the ANR group than in the control group.

**Table 1:** Comparison of characteristics and blood chemistry between the control subjects (CON) and anorexia nervosa patients (AN).

	CON (n = 21)	AN (n = 25)	*p value*	ES (*r*)
Age (years)	31.5 ± 7.4	30.0 ± 10.2	0.7655	0.0439
Height (cm)	158.7 ± 4.5	156.5 ± 4.6	0.1181	0.2304
Weight (kg)	51.7 ± 6.0	31.4 ± 3.6*	**< .0001**	0.8521
BMI (kg/m^2^)	20.5 ± 2.1	12.8 ± 1.3*	**< .0001**	0.8519
Albumin (g/dL)	4.5 ± 0.2	4.1 ± 0.7	0.0262	0.3313
BUN (mg/dL)	12.2 ± 4.0	13.7 ± 6.1	0.4232	0.1194
Crea (mg/dL)	0.62 ± 0.07	0.62 ± 0.15	0.9635	0.0068
Na (mEq/L)	141.5 ± 1.3	140.4 ± 3.9	0.4877	0.1034
K (mEq/L)	4.0 ± 0.2	3.8 ± 0.7	0.4842	0.1042
Cl (mEq/L)	105.2 ± 1.6	101.8 ± 6.9	0.1067	0.2405
Ca (mEq/L)	9.2 ± 0.2	9.1 ± 0.6	0.3897	0.1282
AST (IU/L)	17.8 ± 4.5	38.9 ± 22.9*	**< .0001**	0.6966
ALT (IU/L)	12.7 ± 3.1	40.6 ± 34.0*	**< .0001**	0.6511
T-Chol (mg/dL)	188.5 ± 27.7	208.3 ± 54.0	0.3729	0.1329
TG (mg/dL)	62.0 ± 25.2	83.1 ± 30.1	0.0068	0.4036
Glucose (mg/dL)	82.2 ± 5.7	76.6 ± 11.4	0.0143	0.3651
CRP (mg/dL)	0.08 ± 0.21	0.04 ± 0.06*	**0.0003**	0.5447

BMI, body mass index; BUN, blood urea nitrogen; Crea, creatinine; AST, aspartate aminotransferase; ALT, alanine aminotransferase; T-Chol, total cholesterol; TG, triglycerides; CRP, C-reactive protein; ES, effect size. An asterisk (*) indicates a significant difference between the AN and control group after the Bonferroni correction based on the total number of tests (n = 17, p < 0.0029 (0.05/17)).

**Table 2:** Comparison of characteristics and blood chemistry between the control subjects (CON) and restrictive anorexia nervosa (ANR) or binge-eating anorexia nervosa (ANBP) patients.

	CON (n = 21)	ANR (n = 14)	ANBP (n = 11)	KW^1^	CON vs ANR^2^		CON vs ANBP^2^		ANR vs ANBP^2^	
				*p value*	*p value*	ES (*r*)	*p value*	ES (*r*)	*p value*	ES (*r*)
Age (years)	31.5 ± 7.4	28.1 ± 10.7	32.5 ± 9.4	0.5365	0.4477	0.1283	0.7353	0.0598	0.2721	0.2197
Height (cm)	158.7 ± 4.5	155.1 ± 4.3	158.3 ± 4.5	0.1189	0.0424	0.3431	0.6897	0.0706	0.2053	0.2533
Weight (kg)	51.7 ± 6.0	30.6 ± 4.2*	32.4 ± 2.6*	< .0001	**< .0001**	0.8342	**< .0001**	0.8071	0.4766	0.1424
BMI (kg/m^2^)	20.5 ± 2.1	12.7 ± 1.5*	13.0 ± 1.2*	< .0001	**< .0001**	0.8338	**< .0001**	0.8066	0.848	0.0383
Albumin (g/dL)	4.5 ± 0.2	4.3 ± 0.6	3.8 ± 0.7	0.0162	0.4025	0.1674	0.0024	0.5461	0.1314	0.2587
BUN (mg/dL)	12.2 ± 4.0	14.9 ± 7.3	12.2 ± 3.8	0.4817	0.2851	0.2138	0.8687	0.0284	0.3357	0.1729
Crea (mg/dL)	0.62 ± 0.07	0.59 ± 0.11	0.66 ± 0.20	0.1923	0.2544	0.2047	0.215	0.2126	0.1392	0.2957
Na (mEq/L)	141.5 ± 1.3	142.1 ± 1.6	138.3 ± 4.9	0.0155	0.3241	0.1972	0.0156	0.4343	0.0141	0.4208
K (mEq/L)	4.0 ± 0.2	**4.2 ± 0.4#**	3.2 ± 0.6*	< .0001	0.0484	0.3385	**0.0003**	0.6485	**0.0003**	0.7292
Cl (mEq/L)	105.2 ± 1.6	104.9 ± 2.1	97.8 ± 8.8	0.0208	0.7628	0.0604	0.0111	0.4561	0.0205	0.3974
Ca (mEq/L)	9.2 ± 0.2	9.2 ± 0.6	8.9 ± 0.6	0.3663	0.8878	0.0282	0.1698	0.2466	0.2965	0.179
AST (IU/L)	17.8 ± 4.5	41.6 ± 28.9*	35.5 ± 12.0*	< .0001	**0.0003**	0.6241	**< .0001**	0.8287	1	0
ALT (IU/L)	12.7 ± 3.1	45.2 ± 41.4*	34.7 ± 21.9	< .0001	**< .0001**	0.8028	0.0016	0.5398	0.848	0.0344
T-Chol (mg/dL)	188.5 ± 27.7	189.9 ± 50.7	231.6 ± 50.7	0.0211	0.4008	0.1509	0.0087	0.5247	0.035	0.3616
TG (mg/dL)	62.0 ± 25.2	73.9 ± 28.7	94.8 ± 28.9	0.0078	0.0894	0.2913	0.0041	0.5741	0.0845	0.3098
Glucose (mg/dL)	82.2 ± 5.7	74.3 ± 8.3	79.6 ± 14.3	0.026	0.0052	0.5017	0.2636	0.1917	0.3803	0.1755
CRP (mg/dL)	0.08 ± 0.21	0.02 ± 0.02*	0.06 ± 0.09	0.0001	**< .0001**	0.7913	0.1807	0.2296	0.0341	0.4239

BMI, body mass index; BUN, blood urea nitrogen; Crea, creatinine; AST, aspartate aminotransferase; ALT, alanine aminotransferase; T-Chol, total cholesterol; TG, triglycerides; CRP, C-reactive protein; ES, effect size. An asterisk (*) indicates a significant difference between the AN subgroups and control group after the Bonferroni correction based on the total number of tests (n = 51, p < 0.00098). The sharp (#) indicates a significant difference between the ANR and ANBP groups after the Bonferroni correction (p < 0.00098).

^**1**^ The p value among the ANR, ANBP and control groups was evaluated by the KW (Kruskal-Wallis) test.

^**2**^ Comparisons between two groups were performed using Wilcoxon-Mann-Whitney tests.

As summarized in Table 3, the counts of total bacteria, *Clostridium coccoides* group, *Clostridium leptum* subgroup, *Bacteroides fragilis* group, and *Streptococcus* were significantly lower in the AN groups than in the control group. In the analysis based on AN subtype (Table 4), the counts of the *Bacteroides fragilis* group in the ANR and ANBP groups and the counts of the *Clostridium coccoides* group in the ANR group were significantly lower than in the control group.

**Table 3:** Comparison of bacterial counts between the control subjects (CON) and anorexia nervosa (AN) patients.

	*Log*_*10*_ *cells/g feces*		*p value*	ES (*r*)
	CON (n = 21)	AN (n = 25)		
Total bacteria	11.1 ± 0.5	10.5 ± 0.5*	**0.0002**	0.5560
*C*. *coccoides* group	10.0 ± 0.4	9.3 ± 0.6*	**< .0001**	0.6015
*C*. *leptum* subgroup	10.4 ± 0.7	9.6 ± 0.6*	**0.0006**	0.5138
*B*. *fragilis* group	10.5 ± 0.6	9.6 ± 0.6*	**< .0001**	0.6376
*Bifidobacterium*	10.3 ± 0.7	9.9 ± 1.1	0.1729	0.2055
*Atopobium* cluster	9.3 ± 0.8	9.1 ± 1.2	0.7077	0.0553
*Prevotella*	6.9 ± 1.3	6.4.± 0.8	0.3520	0.1645
*Enterobacteriaceae*	7.1 ± 0.9	7.0 ± 1.0	0.7046	0.0572
*Enterococcus*	6.2 ± 1.2	7.0 ± 1.2	0.0370	0.3144
*Staphylococcus*	5.3 ± 0.9	5.4 ± 0.8	0.9473	0.0098
*Streptococcus*	9.0 ± 0.7	8.2 ± 0.8*	**0.0003**	0.5616
*C*. *difficile*	< 2.4	6.3 ± 1.1	NT	NT
*C*. *perfringens*	4.9 ± 1.3	4.6 ± 1.6	0.5478	0.1281
Total *Lactobacillus*	6.0 ± 1.1	5.7 ± 2.2	0.7065	0.0574
*L*. *gasseri* subgroup	5.4 ± 1.2	5.0 ± 1.8	0.2269	0.2136
*L*. *reuteri* subgroup	4.9 ± 1.0	5.0 ± 1.7	0.8748	0.0394
*L*. *ruminis* subgroup	4.2 ± 1.1	5.8 ± 2.1	0.1123	0.3969
*L*. *plantarum* subgroup	4.0 ± 0.8	3.5 ± 1.1	0.3421	0.2179
*L*. *sakei* subgroup	4.4 ± 1.3	3.9 ± 0.6	0.4743	0.1461
*L*. *casei* subgroup	5.8 ± 1.4	6.5 ± 1.4	0.3787	0.1921
*L*. *brevis*	5.3 ± 0.3	4.2 ± 0.4	0.2453	0.5810
*L*. *fermentum*	4.6 ± 0.7	8.7	0.2416	0.4781

*C*, *Clostridium; B*, *Bacteroides; L*, *Lactobacillus;*ES, effect size. NT means "not tested" because at least one group is below detection limits. The total count of *Lactobacillus* obtained by YIF-SCAN® is expressed as the sum of the counts of 6 *Lactobacilli* subgroups and 2 species. An asterisk (*) indicates a significant difference between the AN and control group after the Bonferroni correction based on the number of tests (n = 21, p < 0.0024 (0.05/21)).

**Table 4:** Comparison of bacterial counts between the control subjects (CON) and restrictive anorexia nervosa (ANR) or binge-eating anorexia nervosa (ANBP) patients.

	*Log*_*10*_ *cells/g feces*			KW^1^	CON vs ANR^2^		CON vs ANBP^2^		ANR vs ANBP^2^	
	CON (n = 21)	ANR (n = 14)	ANBP (n = 11)	*p value*	*p value*	ES (*r*)	*p value*	ES (*r*)	*p value*	ES (*r*)
Total bacteria	11.1 ± 0.5	10.4 ± 0.5	10.6 ± 0.5	0.0006	0.001	0.5549	0.0038	0.512	0.2857	0.2135
*C*. *coccoides* group	10.0 ± 0.4	9.3 ± 0.6*	9.2 ± 0.6	0.0002	**0.0003**	0.6118	0.0023	0.5401	0.7633	0.06
*C*. *leptum* subgroup	10.4 ± 0.7	9.6 ± 0.6	9.7 ± 0.6	0.0024	0.0023	0.5151	0.0106	0.4592	0.7474	0.0657
*B*. *fragilis* group	10.5 ± 0.6	9.6 ± 0.7*	9.5 ± 0.6*	0.0001	**0.0004**	0.5948	**0.0007**	0.6197	0.7768	0.0591
*Bifidobacterium*	10.3 ± 0.7	9.5 ± 1.2	10.2 ± 0.8	0.1124	0.0414	0.3551	0.9053	0.021	0.1481	0.3016
*Atopobium* cluster	9.3 ± 0.8	9.4 ± 0.5	8.6 ± 1.6	0.3911	0.7747	0.0484	0.3023	0.1824	0.1798	0.2683
*Prevotella*	6.9 ± 1.3	6.5 ± 0.8	6.1 ± 0.8	0.3883	0.8458	0.0381	0.1717	0.279	0.4014	0.2243
*Enterobacteriaceae*	7.1 ± 0.9	6.7 ± 1.1	7.3 ± 0.7	0.4939	0.3722	0.1554	0.7306	0.0629	0.2857	0.2135
*Enterococcus*	6.2 ± 1.2	6.6 ± 1.0	7.6 ± 1.3	0.0204	0.3667	0.1571	0.0088	0.4709	0.0489	0.4021
*Staphylococcus*	5.3 ± 0.9	5.4 ± 0.9	5.4 ± 0.7	0.969	0.9597	0.0085	0.8427	0.0345	0.848	0.0383
*Streptococcus*	9.0 ± 0.7	8.3 ± 0.8	8.0 ± 0.7	0.0007	0.007	0.4844	0.0009	0.605	0.1858	0.2759
*C*. *difficile*	< 2.4	< 2.4	6.3 ± 1.1	NT	NT	NT	NT	NT	NT	NT
*C*. *perfringens*	4.9 ± 1.3	4.8 ± 1.3	4.3 ± 2.0	0.64	1	0	0.3358	0.2334	0.7133	0.1225
Total *Lactobacillus*	6.0 ± 1.1	4.8 ± 1.9	6.6 ± 2.2	0.0645	0.0742	0.3108	0.2499	0.2003	0.0488	0.42
*L*. *gasseri* subgroup	5.4 ± 1.2	4.1 ± 1.1	5.7 ± 2.0	0.0783	0.0262	0.4537	0.8842	0.0291	0.1182	0.4034
*L*. *reuteri* subgroup	4.9 ± 1.0	3.4 ± 0.6	5.5 ± 1.7	0.1933	0.151	0.4541	0.6514	0.1208	0.1336	0.5303
*L*. *ruminis* subgroup	4.2 ± 1.1	4.4 ± 2.7	6.6 ± 1.4	0.058	0.8197	0.072	0.0184	0.7857	0.2453	0.3223
*L*. *plantarum* subgroup	4.0 ± 0.8	3.2 ± 0.7	3.9 ± 1.6	0.2798	0.1175	0.3796	0.9406	0.0181	0.6985	0.1937
*L*. *sakei* subgroup	4.4 ± 1.3	3.9 ± 0.8)	3.9 ± 0.4	0.7504	0.6005	0.1171	0.5823	0.1262	0.9025	0.0408
*L*. *casei* subgroup	5.8 ± 1.4	6.2 ± 1.2	6.7 ± 1.5	0.5868	0.832	0.0567	0.3055	0.2485	0.7768	0.0855
*L*. *brevis*	5.3 ± 0.3	< 2.6	4.2 ± 0.4	NT	NT	NT	0.2453	0.5810	NT	NT
*L*. *fermentum*	4.6 ± 0.7	< 4.0	8.7	NT	NT	NT	0.2416	0.4781	NT	NT

*C*, *Clostridium; B*, *Bacteroides; L*, *Lactobacillus*; ES, effect size. NT, "not tested" because at least one group is below detection limits. The total count of *Lactobacillus* obtained by YIF-SCAN® is expressed as the sum of the counts of 6 *lactobacilli* subgroups and 2 species. An asterisk (*) shows a significant difference between the AN subgroup and the control group after application of the Bonferroni correction based on the total number of tests (n = 59, p < 0.00084 (0.05/59)).

^**1**^ Statistical differences between the ANR, ANBP, and control groups were evaluated using the KW (Kruskal-Wallis) test.

^**2**^ Comparisons between two groups were made using the Wilcoxon-Mann-Whitney test.

As shown in Table 5, the detection rate of the *Lactobacillus plantarum* subgroup was significantly lower in the AN group than in the control group. Interestingly, 5 of 11 (45%) ANBP patients had *Clostridium difficile* in their feces, while neither the control nor ANR patients had any detectable levels of this pathogen (Table 6); however, the differences between the groups (Fisher’s exact tests, control vs. ANBP, p = 0.0023; ANR vs. ANBP, p = 0.0087) were not statistically significant after the Bonferroni correction.

**Table 5:** Comparison of bacterial prevalence between the control (CON) subjects and anorexia nervosa (AN) patients.

	CON (n = 21)		AN (n = 25)		
	Number of positive samples	Detection rate (%)	Number of positive samples	Detection rate (%)	*p value* ^*1*^
Total bacteria	21	100	25	100	NT
*C*. *coccoides group*	21	100	25	100	NT
*C*. *leptum subgroup*	21	100	24	96	1
*B*. *fragilis group*	21	100	23	100	NT
*Bifidobacterium*	21	100	23	92	0.4928
*Atopobium cluster*	21	100	25	100	NT
*Prevotella*	18	86	14	56	0.0521
*Enterobacteriaceae*	19	90	25	100	0.2029
*Enterococcus*	20	95	24	96	1
*Staphylococcus*	21	100	25	100	NT
*Streptococcus*	19	90	23	92	1
*C*. *difficile*	0	0	5	20	0.0536
*C*. *perfringens*	13	62	9	36	0.1378
*Total Lactobacillus*	21	100	22	88	0.2391
*L*. *gasseri subgroup*	17	81	15	60	0.1988
*L*. *reuteri subgroup*	8	38	8	32	0.7604
*L*. *ruminis subgroup*	7	33	9	36	1
*L*. *plantarum subgroup*	15	71	4	16*	**0.0002**
*L*. *sakei subgroup*	15	71	9	36	0.0210
*L*. *casei subgroup*	10	48	11	44	1
*L*. *brevis*	2	10	2	8	1
*L*. *fermentum*	5	24	2	8	0.0790

*C*, *Clostridium; B*, *Bacteroides; L*, *Lactobacillus*. NT, "not tested" because of no difference in the bacterial detection rate between the two groups. Comparisons between the two groups were conducted using Fisher's exact test followed by the Bonferroni correction. An asterisk (*) indicates a significant difference between the AN and control groups after the Bonferroni correction based on the total number of tests (n = 17, p < 0.0029 (0.05/17)).

**Table 6:** Comparison of bacterial prevalence between the control (CON) subjects and restrictive anorexia nervosa (ANR) or binge-eating anorexia nervosa (ANBP) patients.

	CON (n = 21)		ANR (n = 14)		ANBP (n = 11)		Con vs ANR^1^	Con vs ANBP^1^	ANR vs ANBP^1^
	Number of positive samples	Detection rate (%)	Number of positive samples	Detection rate (%)	Number of positive samples	Detection rate (%)	*p value*	*p value*	*p value*
Total bacteria	21	100	14	100	11	100	NT	NT	NT
*C*. *coccoides group*	21	100	14	100	11	100	NT	NT	NT
*C*. *leptum subgroup*	21	100	14	100	10	91	NT	0.3438	0.44
*B*. *fragilis group*	21	100	14	100	9	82	NT	0.1109	0.1833
*Bifidobacterium*	21	100	12	86	11	100	0.1529	NT	0.4867
*Atopobium cluster*	21	100	14	100	11	100	NT	NT	NT
*Prevotella*	18	86	8	57	6	55	0.1122	0.0877	1
*Enterobacteriaceae*	19	90	14	100	11	100	0.5059	0.5343	NT
*Enterococcus*	20	95	13	93	11	100	1	1	1
*Staphylococcus*	21	100	14	100	11	100	NT	NT	NT
*Streptococcus*	19	90	12	86	11	100	1	0.5343	0.4867
*C*. *difficile*	0	0	0	0	5	45	NT	0.0023	0.0087
*C*. *perfringens*	13	62	5	36	4	36	0.1756	0.2662	1
*Total Lactobacillus*	21	100	11	79	11	100	0.0556	NT	0.23
*L*. *gasseri subgroup*	17	81	7	50	8	73	0.0725	0.6675	0.4139
*L*. *reuteri subgroup*	8	38	2	14	6	55	0.2516	0.4651	0.081
*L*. *ruminis subgroup*	7	33	3	21	7	64	0.7041	0.2826	0.1153
*L*. *plantarum subgroup*	15	71	2	14	2	18	0.0016	0.008	1
*L*. *sakei subgroup*	15	71	5	36	4	36	0.0796	0.0721	1
*L*. *casei subgroup*	10	48	4	29	7	64	0.3109	0.4719	0.116
*L*. *brevis*	2	10	0	0	2	18	0.5059	0.5932	0.1833
*L*. *fermentum*	5	24	0	0	1	9	0.0689	0.6367	0.44

*C*, *Clostridium; B*, *Bacteroides; L*, *Lactobacillus*. NT, "not tested" because of no difference in bacterial detection rate between the two groups.

^**1**^ Comparisons between the two groups were made using the Fisher's exact test followed by the Bonferroni correction based on the number of total tests (n = 47).

As summarized in Table 7, the AN group had significantly lower fecal concentrations of acetic acid and propionic acid than the control group. In the AN subtype analysis (Table 8), the acetic acid levels were significantly lower in the ANR group than in the control group.

**Table 7:** Comparison of organic acids and pH between the control subjects (CON) and anorexia nervosa (AN) patients.

	*μmol/g feces*			
	Controls (n = 21)	AN (n = 25)	*p value*	ES (*r*)
Total organic acids	87.9 ± 43.9	54.3 ± 20.6	0.0049	0.4246
Succinic acid	5.9 ± 10.6	16.4 ± 18.0	0.3506	0.2200
Lactic acid	11.2 ± 12.8	0.9	0.2888	0.4743
Formic acid	0.1 ± 0.0	2.27 ± 2.71	0.0265	0.6405
Acetic acid	58.6 ± 27.0	30.7 ± 13.2*	**0.0003**	0.5455
Propionic acid	15.2 ± 5.9	9.3 ± 4.8*	**0.0010**	0.4957
Iso-butyric acid	1.2 ± 1.4	13.6 ± 8.2	0.0591	0.6674
Butyric acid	9.6 ± 7.5	3.8 ± 2.5	0.0082	0.5089
Isovaleric acid	3.1 ± 2.2	4.3 ± 1.4	0.18	0.3583
Valeric acid	4.2	4.7 ± 1.2	1	0
pH	6.74 ± 0.94	7.37 ± 0.86	0.0374	0.3103

ES: effect size. Total organic acid concentration is expressed as the sum of the concentrations of 9 acids. An asterisk (*) indicates a significant difference between the AN and control groups after a the Bonferroni correction based on the total number of tests (n = 11, p < 0.0045 (0.05/11)).

**Table 8:** Comparison of organic acids and pH between the control subjects (CON) and restrictive anorexia nervosa (ANR) or binge-eating anorexia nervosa (ANBP) patients.

	*μmol/g feces*			KW^1^	CON vs ANR^2^		CON vs ANBP^2^		ANR vs ANBP^2^	
	Controls (n = 21)	ANR (n = 14)	ANBP (n = 11)	*p value*	*p value*	ES (*r*)	*p value*	ES (*r*)	*p value*	ES (*r*)
Total organic acids	87.9 ± 43.9	49.8 ± 18.4	55.6 ± 23.3	0.0159	0.0084	0.4586	0.0499	0.3522	0.6021	0.1064
Succinic acid	5.9 ± 10.6	16.1 ± 23.6	16.5 ± 16.9	0.3979	0.2026	0.2218	0.9468	0.012	0.2845	0.2185
Lactic acid	11.2 ± 12.8	0.9	ND	0.2011	0.3015	0.1799	0.1277	0.2736	0.403	0.1707
Formic acid	0.1 ± 0.0	1.68 ± 1.89	2.7 ± 3.4	0.0877	0.1976	0.2243	0.0298	0.3903	0.4241	0.1632
Acetic acid	58.6 ± 27.0	28.0 ± 11.6*	33.8 ± 14.8	0.001	**0.0007**	0.5869	0.0111	0.456	0.339	0.1952
Propionic acid	15.2 ± 5.9	10.0 ± 4.8	8.3 ± 4.8	0.0031	0.0104	0.4458	0.0041	0.5154	0.2584	0.2307
Iso-butyric acid	1.2 ± 1.4	17.5 ± 0.7	9.7 ± 11.8	0.9896	0.9345	0.0143	0.9761	0.0054	0.9289	0.0182
Butyric acid	9.6 ± 7.5	3.2 ± 2. 2	4.7 ± 3.1	0.0041	0.006	0.4781	0.0094	0.4665	0.6125	0.1034
Isovaleric acid	3.1 ± 2.2	5.0 ± 1.3	3.5 ± 1.2	0.4645	0.2669	0.1933	0.4398	0.1387	0.5936	0.1089
Valeric acid	4.2	4.7 ± 1.2	ND	0.1125	0.1436	0.2546	0.5002	0.1211	0.1066	0.3294
pH	6.74 ± 0.94	7.38 ± 0.91	7.35 ± 0.83	0.1104	0.0825	0.2978	0.0956	0.2946	0.772	0.0591

ND: not detected. Total organic acid concentration is expressed as the sum of the concentrations of 9 acids. An asterisk (*) indicates a significant difference between the AN subgroup and the control group after application of the Bonferroni correction based on the total number of tests (n = 33).

^**1**^ Statistical differences between the ANR, ANBP, and control groups were evaluated using the KW (Kruskal-Wallis) test.

^**2**^ Comparisons between the two groups were performed using the Wilcoxon-Mann-Whitney test followed by the Bonferroni correction based on the number of groups of subjects (n = 33, p < 0.0015 (0.05/33)).

**Fig 1:**
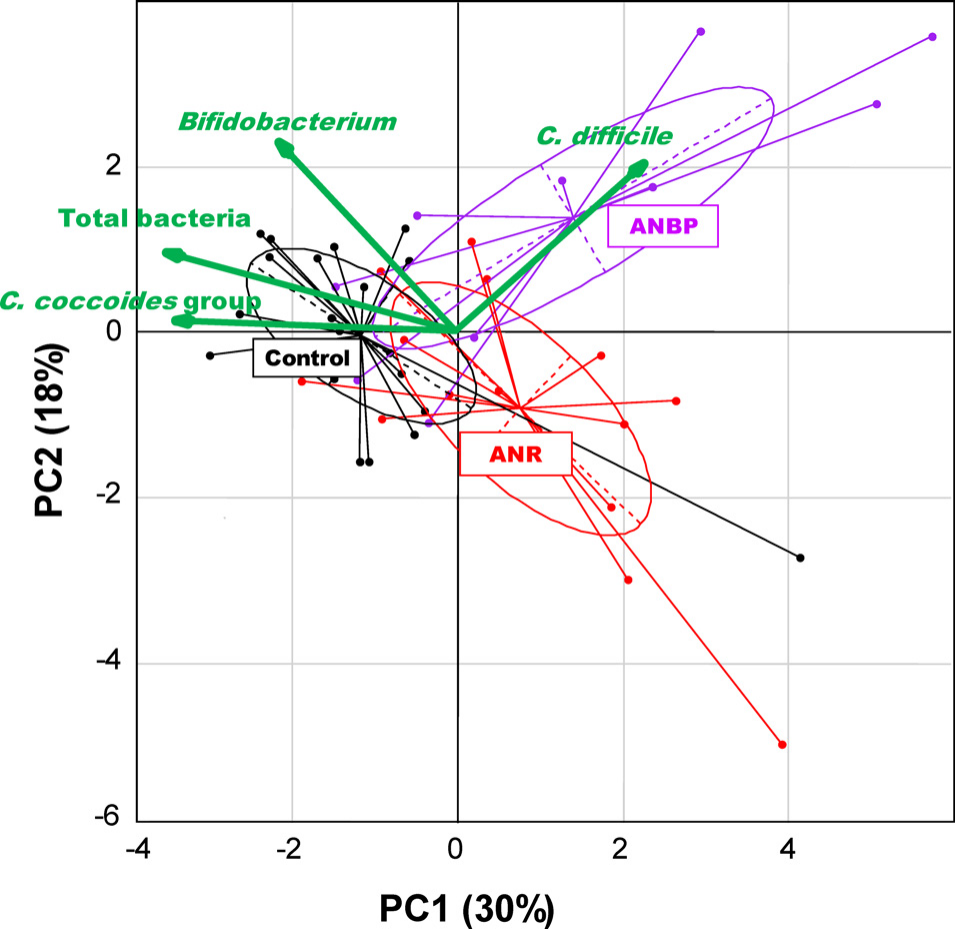
Principal component analysis (PCA) of bacterial counts in healthy female controls, 14 restrictive anorexia nervosa (ANR) patients, and 10 binge-eating anorexia nervosa (ANBP) patients. Black, red, and purple plots show data for the healthy female controls, ANR patients, and ANBP patients, respectively. The colored ellipse represents 50% of the samples. Arrows indicate the characteristic vectors of the upper 4 factor loadings. The numbers in parentheses represent the proportion of variance.

The differences in bacterial components between the groups were also supported by the PCA results (Fig 1). In particular, a clear separation was observed between the plots for AN patients and control women along the PC1 axis, which had highly negative correlations with the counts of *C*. *coccoides* and total bacteria.

## Discussion

In the current study, AN patients had significantly lower amounts of total bacteria, *C*. *coccoides* group, *C*. *leptum* subgroup, *B*. *fragilis*, and *Streptococcus* than age-matched healthy women. In addition, acetic acid and propionic acid levels were significantly lower in the AN group than in the control group. The detection rate of the *Lactobacillus plantarum* subgroup was also significantly lower in the AN group than in the control group. In the AN subtype analysis, the counts of the *Bacteroides fragilis* group in both the ANR and ANBP groups and the counts of the *Clostridium coccoides* in the ANR group were significantly lower than those in the control group. The PCA results showed that the patterns of gut microbiota in the AN group were different from those in the control group. Collectively, these results indicate that microbial imbalance, i.e. dysbiosis, does exist in the gut of AN patients.

The importance of gut microbes in weight control was first reported as a beneficial effect of antibiotics on growth promotion in poultry [26, 27]. Antibiotic growth promotion in the livestock industry has since been practiced in the United States and other countries. Because oral antibiotics failed to exert any growth-promoting effects on germ-free animals [28], the mechanism for such growth promotion is thought to be mediated by gut microbiota. Recently, this concept was further advanced by a series of excellent work by Gordon and colleagues [15–17], who demonstrated that obesity is linked with changes in the relative abundance of the two dominant bacterial divisions of the gut microbiome, *Bacteroidetes* and *Firmicutes*. These findings clearly demonstrate the importance of gut microbes in the regulation of weight gain.

In a recent report from a group in France, higher amounts of *Methanobrevibacter smithii*, a predominant archaeon in the human gut, were found in nine anorexic patients than in lean patients [29]. More recently, an exciting paper demonstrated that transplantation of *Christensenella minuta*, a cultured member of the *Christensenellaceae* family, to germ-free mice reduced weight gain by altering the microbiome of recipient mice [30]. Although those microorganisms were not included in the analysis in the present study, these findings collectively suggest that gut dysbiosis or a certain bacterium found in AN patients might be a causal factor for maintenance of emaciation. To solve this important and fundamental question, we have a new project in progress to unravel a comprehensive feature of the AN-specific gut microbiome using a next generation sequencer, in combination with animal studies using gnotobiotic mice reconstituted with AN gut microbes.

In this study, *C*. *difficile* was only detected in the ANBP group, but not in the control and ANR groups. Several risk factors are implicated in the establishment of *C*. *difficile* infection [31]. Exposure to antimicrobial agents, the most well known risk factor, increases the possibility of *C*. *difficile* infection because it disturbs the composition of gut microbiota. The use of acid-suppressing medication such as histamine type 2 receptor blockers and proton pump inhibitors is also suggested as another potential risk factor [32–34]; however, an epidemiologic association between the use of stomach acid-suppressing medications and *C*. *difficile* infection is still inconclusive [31, 35–37]. In the present study, all of the AN patients did not take any antibiotics during the previous three months. In addition, one of five *C*. *difficile*-positive ANBP patients had a prescription for rabeprazole, while the rest were not taking any stomach acid-suppressing medications, indicating no direct association between the use of antibiotics or stomach acid-suppressing agents and the development of *C*. *difficile* infection in our patients. Because ANBP patients often have esophageal and gastric abnormalities due to frequent vomiting, these results suggest ANBP-specific behaviors, such as recurrent purging, to be a possible risk factor for *C*. *difficile* colonization.

Molecular biology methods have been developed recently to study microbiota by targeting rRNA genes. The YIF-SCAN^®^ was developed based on RT-qPCR using specific primers that target bacterial rRNA molecules [20, 23]. By targeting rRNA molecules, the YIF-SCAN^®^ has 100–1,000 times the sensitivity of conventional PCR methods, enabling microbiota analysis with a strikingly wide dynamic range. Indeed, the detection of *C*. *difficile* in this study was made possible because of its high level of sensitivity.

This study has several limitations. First, we cannot draw conclusions about a causal link between gut dysbiosis and difficulty in weight gain because of the cross-sectional study design. This problem can be addressed using gnotobiotic mice which have human AN-specific microbiota. Second, the YIF-SCAN^®^ only covers species of bacteria that can be detected with a specific primer. Third, the sample size was relatively small. This may mask some of the true variation in the population, and may contribute to the perception of differences following multi-dimensional reduction. Therefore, the current results should be confirmed in future studies with a large sample size.

In conclusion, the current results indicate the presence of gut dysbiosis in AN patients. Unraveling the specific details of an AN-specific gut microbiome might be useful in developing a therapeutic option for this debilitating disorder.

## Supporting Information

S1 Table Sequences of the primers and detection limits of each bacterium. (PDF)
