# Supplementary material for: Gut Dysbiosis in Patients with Anorexia Nervosa
Source: PLoS One. 2015 Dec 18;10(12):e0145274. doi: 10.1371/journal.pone.0145274 (PMC4687631; doi:10.1371/journal.pone.0145274)
Supplement: S1 Table — (PDF) [file pone.0145274.s001.pdf]

S1 Table. Sequences of the primers and detection limits of each bacterium

Target bacteria	Primer	Sequence (5' - 3')	Detection limits (Log10/g feces)	Reference No.
Clostridium coccoides group	g-Ccoc-F	AAATGACGGTACCTGACTAA	5.0	1
	g-Ccoc-R	CTTTGAGTTTCATTCTTGCGAA		
Clostridium leptum subgroup	sg-Clept-F	GCACAAGCAGTGGAGT	5.0	1
	sg-Clept-R3	CTTCCTCCGTTTTGTCAA		
Bacteroides fragilis group	g-Bfra-F2	AYAGCCTTTCGAAAGRAAGAT	5.0	2
	g-Bfra-R	CCAGTATCAACTGCAATTTTA		
Bifidobacterium	g-Bifid-F	CTCCTGGAAACGGGTGG	5.0	3
	g-Bifid-R	GGTGTTCTTCCCGATATCTACA		
Atopobium cluster	g-Atopo-F	GGGTTGAGAGACCGACC	5.9	1
	g-Atopo-R	CGGRGCTTCTTCTGCAGG		
Prevotella	g-Prevo-F	CACRGTAAACGATGGATGCC	5.1	1
	g-Prevo-R	GGTCGGGTTGCAGACC		
Enterobacteriaceae	En-lsu-3F	TGCCGTAACTTCGGGAGAAGGCA	3.9	7
	En-lsu-3'R	TCAAGGACCAGTGTTCAGTGTC		
Enterococcus	g-Encoc-F	ATCAGAGGGGGATAACACTT	3.0	5
	g-Encoc-R	ACTCTCATCCTTGTTCTTCTC		
Staphylococcus	g-Staph-F	TTTGGGCTACACACGTGCTACAATGGACAA	3.0	5
	g-Staph-R	AACAACTTTATGGGATTTGCWTGA		
Streptococcus	g-Str-F	AGCTTAGAAGCAGCTATTCATTC	3.0	8
	g-Str-R	GGATACACCTTTCGGTCTCTC		
Clostridium difficile	Cd-lsu-F	GGGAGCTTCCCATACGGGTTG	2.4	4
	Cd-lsu-R	TTGACTGCCTCAATGCTTGGGC		
Clostridium perfringens	s-Clper-F	GGGGGTTTCAACACCTCC	2.3	5
	ClPER-R	GCAAGGGATGTCAAGTGT		6
Lactobacillus gasseri subgroup	sg-Lgas-F	GATGCATAGCCGAGTTGAGAGACTGAT	2.7	5
	sg-Lgas-R	TAAAGGCCAGTTACTACCTCTATCC		
Lactobacillus reuteri subgroup	sg-Lreu-F	GAACGCAYTGGCCCAA	3.0	5
	sg-Lreu-R	TCCATTGTGGCCGATCAGT		
Lactobacillus ruminis subgroup	sg-Lrum-F	CACCGAATGCTTGCAYTCACC	2.3	5
	sg-Lrum-R	GCCGCGGGTCCATCCAAAA		
Lactobacillus plantarum subgroup	sg-Lpla-F	CTCTGGTATTGATTGGTGCTTGCAT	2.6	5
	sg-Lpla-R	GTTCGCCACTCACTCAAATGTAAA		
Lactobacillus sakei subgroup	sg-Lsak-F	CATAAAACCTAMCACCGCATGG	2.9	5
	sg-Lsak-R	TCAGTTACTATCAGATACRTTCTTCTC		
Lactobacillus casei subgroup	sg-Lcas-F	ACCGCATGGTTCTTGGC	3.5	5
	sg-Lcas-R	CCGACAACAGTTACTCTGCC		
Lactobacillus brevis	s-Lbre-F	ATTTTGTTTGAAAGGTGGCTTCGG	2.6	5
	s-Lbre-R	ACCCTTGAACAGTTACTCTCAAAGG		
Lactobacillus fermentum	LFer-1	CCTGATTGATTTTGGTCGCCAAC	4.0	5
	LFer-2	ACGTATGAACAGTTACTCTCATACGT		

References

1. Matsuki T, Watanabe K, Fujimoto J, et al. Use of 16S rRNA gene-targeted group-specific primers for real-time PCR analysis of predominant bacteria in human feces. Appl Environ Microbiol. 2004;70(12):7220-7228.

2. Matsuki T. Development of quantitative PCR detection method with 16S rRNA gene-targeted genus- and species-specific primers for the analysis of human intestinal microflora and its application. Nippon Saikingaku Zasshi. 2007; 62(2): 255-261

3. Matsuki T. Phylogenetic Relationship of Predominant Bacteria in Human Intestinal Bacteria Based on the 16S rRNA Gene Sequence. J Intestinal Microbiol. 2008;22: 253-261

4. Matsuda K, Tsuji H, Asahara T, Takahashi T, Kubota H, Nagata S, Yamashiro Y, Nomoto K. Sensitive quantification of Clostridium difficile cells by reverse transcription-quantitative PCR targeting rRNA molecules. Appl Environ Microbiol. 2012 Aug;78(15):5111-8.

5. Matsuda K, Tsuji H, Asahara T, Kado Y, Nomoto K. Sensitive quantitative detection of commensal bacteria by rRNA-targeted reverse transcription-PCR. Applied and environmental microbiology 2007; 73(1): 32-39.

6. Kikuchi E, Miyamoto Y, Narushima S, et al. Design of species-specific primers to identify 13 species of Clostridium harbored in human intestinal tracts. Microbiol Immunol. 2002;46(5):353–358.

7. Matsuda K, Tsuji H, Asahara T, Matsumoto K, Takada T, Nomoto K. Establishment of an analytical system for the human fecal microbiota, based on reverse transcription-quantitative PCR targeting of multicopy rRNA molecules. Applied and environmental microbiology 2009;

8. Sakaguchi S, Saito M, Tsuji H, Asahara T, Takata O, Fujimura J, Nagata S, Nomoto K, Shimizu T. Bacterial rRNA-targeted reverse transcription-PCR used to identify pathogens responsible for fever with neutropenia. J Clin Microbiol 2010;48(5):1624-8.
